# Dr. Green’s Observations on the Treatment of Eczema

**Published:** 1842-10-01

**Authors:** Jonathan Green


*Some Observations on the Treatment of Eczema.* By JONATHAN GREEN, M. D., M. R. C. S., &c.—The treatment of eczema is, for the most part, so uncertain and unsatisfactory, that I trust a few remarks on the subject may not be without interest, as they are the result of frequent practical observation. The symptoms of the disease itself it is needless to particularise; they vary according to the extent, the region, the degree, or stage of the malady, and the serous discharge is always characteristic of the fully-developed state of the disease, to which I would draw attention.

It is always readily known by the scalded appearance of the parts affected; mostly the legs, the thighs, and arms of elderly persons are the seats of these procrastinated ailments; but no region of the body seems to be exempt from the disease, and it often attacks the flexures of the joints, scrotum, &c., to the great inconvenience of the patient, though otherwise not often of a serious nature. The sudden and profuse quantity of clear watery discharge that attends eczema will always characterise the disease; and this is often so abundant, as to require doubled and quadrupled folds of sheets, or other covering to absorb it, and these frequently require to be renewed. It must be evident that I am now alluding to aggravated cases, those which are alike commonly treated with dissatisfaction to the practitioner and patient. Applications, even of the most bland description, as simply of oil, lard, ablutions of bran water, of warm water alone, or combined with the various preparations of lead, opium, &c., from which sedative effects might be expected; yet they seem for the most part to be of little avail in severe cases of the disease. The general health of the patient, however, is often not in the least interrupted; the various internal functional operations seem to proceed with little or no deviation from that which constitutes a state of health. Sometimes the tongue is covered in part or on its whole surface with a brown or yellow thick mucus; the urine small in quantity, clouded, and depositing reddish sediment, and the pulse is quick and variable. When these latter symptoms attend the disorder, appropriate treatment must be had recourse to as indicated, for persons may have at the same time various other ailments conjoined with the eczema, but, generally speaking, for the latter, internal treatment would seem to be uncalled for.

The disease is usually attended with much anxiety and irritability, and an alarm, which is not borne out by the serious implication or derangement of any of the more important organs of the body threatening danger to the patient's life; and however general eczema may be over the patient's body, it usually gets well after a period of more or less duration and perplexity of management. The sulphur fumigating baths, so much extolled on the continent for the treatment of cutaneous disorders, are of no good avail in the treatment of eczema, but mischievous, unless resorted to at the latter stages of the disease, and administered in a very qualified way only to be learnt by practice with this important means of relief. This observation may be applied with equal truth as regards these baths, when applied to all the vesicular and prurigonous disorders of the skin in their early or active stages. The diseases last alluded to require the fumigations to be administered at a low temperature, and in a modified manner, and at the stages of their decadence, if we would calculate on benefit from their use. With this reserve I fully agree with all that has been advocated for the utility of the fumigations.

On the legs, thighs, and arms, eczema is mostly to be found on those who labour under the malady when in an aggravated state. The parts appear denuded of the cuticle, as though they had been scalded, and the opening of the capillary exhalents are continuously exuding such a quantity of serous fluid, as to quite surprise the patient and the practitioner not accustomed to the disease. This discharge continues as long as the disease remains in an acute state, rebellious to all the customary and well-considered means resorted to for its amendment. The parts affected will continue discharging serum for weeks, and sometimes for months, going through all dressings, and even sometimes through the bedding. Proper absorbing substances are in constant requisition, the edges or folds of which become stiff and adhering, and their removal occasions excoriations more or less deep and bleeding; the latter, however, is never to much extent, and seems to do no harm. These frequent dressings have appeared to me a source of evil, by prolonging the disorder; yet it is not often, even in the state above named, that there is any imperious call for internal medical aid; the bodily functions go on well, with the exception of the skin, nor does it seem advisable that the patient should be put on too low a dietary system; the serous discharge so profusely escaping, would appear to prevent any high degree of internal inflammatory symptoms from taking place. A moderate diet with quiet, and the bowels perfectly free, is all that seems absolutely needful to be observed; in robust young subjects a moderate bleeding may be essayed, and other indications appropriately met.

Without entering into detail as regards various remedial means, I at once say, that after repeatedly trying the usual routine of medicines and applications in the treatment of eczema, generally with disappointment, and seeing the health of the patient commonly so little disturbed, led me to direct my attention principally to external means for relief. With a view of abating the apparent high state of superficial inflammation in eczema, I have used the extract of lead to great extent; equal parts of grease, or cream, and of the extract, have been applied all over very extensive surfaces of eczema: I

am not aware that I ever did any good with this application, but I name it in order to say I have never known any evil to follow its application; this is contrary to general assent; perhaps the exemption from evil may have been owing to the fumigating baths being used at the same time, as the latter occasions an excess of action in the exhalents of the system, dependent on the view of the prescriber, and on the way of administering this fumigation; but certain it is, I have used the said extract with little regard as to quantity, and without ill effects, and after a time I ceased to apprehend any ill effects from its use. Without detailing the various means which ended in disappointment, I at once proceed to the mode that has answered best with me in treating eczema. It is to keep the patient as still and quiet as possible, subjected to a moderate warmth, not to produce sweating, but only to keep insensible perspiration active. I cover all the diseased surface with an ointment composed of a drachm of calcined magnesia, rubbed into two ounces of melted lard (the lard when previously melted takes up more readily the magnesia,) and this mixture also admits better of any additional medicament that may be desired. This sort of ointment is scarcely greasy to the feel, and I have found it much better than the chalk ointment frequently used; the latent carbonic acid and lime contained in the chalk does not agree with the irritable diseased surface of eczema. This magnesia ointment being a little warmed, is smeared all over the eczematous surface, and then covered with tissue paper; and the longer it can be allowed to remain without removal the better. At first it may require to be renewed once, or even twice a-day; this is easily done: any parts of the tissue paper which are not positively pushed off by the discharge are allowed to remain; and where the discharge has broken through, and as it were washed away the paper, a fresh smearing of the absorbing magnesia ointment is applied, and a patch or many patches of the tissue paper, according to the extent of surface, is put over it as before. In two, three, or four days, quiet being observed in the interval, the discharge of the eczema becomes greatly lessened, ceasing to push off the paper, yet in places it may be felt fluctuating under it: no matter, as long as the paper remains entire, I do not remove it for the sake of redressing, which I only resort to, to parts or places where it is absolutely necessary. I am convinced that the less we interfere either externally or internally with eczema, the better it is, and the sooner we get over the high excitement of this apparently formidable disease on the surface. Whilst this magnesia application and the paper will remain on, it is well to let it do so without removal; it will become black in places, attended with some little fætor; but if the latter is not much in degree, I suffer it to remain until the paper falls off of itself, and the parts under it are generally found to be healing, or quite healed; if not the latter, another dressing as before is applied, and allowed to remain, and thus repeated as long as needed. It is surprising how soon the eczema will heal under this method; cases that have previously required incessant attention, and no amendment after months of treatment, often get well in three or four weeks. The cuticle will then continue to throw off abundance of squamous laminæ, and it is at this period that the mild fumigation may be brought to aid with great advantage, or the simple vapour bath may be used; but if either one or the other is used previous to the decadence of the disease, they do no good, but the contrary. Greasy applications, indeed, all applications are, for the most part, unsatisfactory in eczema; I would except this magnesian ointment, which is hardly to be called greasy, and, according to the mode just recommended of using it has answered better with me than any other mode of treating eczema. Nevertheless, I am of opinion that much of the amendment that follows is due to the quiet and little interference resorted to after this method of treating the disease. It seems to hasten and bring the thin acrimonious discharge into that of a bland and pus-like nature. As long as the thin ichorous discharge continues, there is no end to the trouble, and the surface implicated goes on extending. Attention to cleanliness, by washing with lotions, water, thin gruel, &c., is mostly needless, does no good, and has appeared to me to be a source of aggravation to the ailment under consideration. Internal treatment must depend on any functional derangement that may coexist with the eczema; but very often no derangement of function can be discovered, except that which constitutes the disease itself on the skin. I would add that mercurial medicines appear to act mischievously, even as is known to the producing the disease. The fumigating baths are only admissible when the disease is on the decline, and the squamous form set in with little or no discharge, or when the surface looks of a dark red or purplish colour; the effects of such baths are then of great avail, and where they cannot be had, the use of a simple vapour bath may be substituted with good effect. Bad cases of eczema are of such frequent occurrence, are subject to such sudden accessions without appreciable cause, and so perplexing to control, that I trust these remarks may be of some practical use, as they are the result of daily observation in the management of this disease.—*London Lancet*, Aug. 13, 1842.

